# Surface Coating for Flame Retardancy and Pyrolysis Behavior of Polyester Fabric Based on Calcium Alginate Nanocomposites

**DOI:** 10.3390/nano8110875

**Published:** 2018-10-25

**Authors:** Zhenhui Liu, Jiao Li, Xihui Zhao, Zichao Li, Qun Li

**Affiliations:** 1 College of Chemistry and Chemical Engineering, Qingdao University, Qingdao 266071, China; 2016020405@qdu.edu.cn (Z.Liu); lijiao201500@163.com (J.L.); zhaoxihui@qdu.edu.cn (X.Z.); 2 College of Life Sciences, Qingdao University, Qingdao 266071, China

**Keywords:** polyester fabric, calcium alginate composites, flame retardancy, thermal properties, anti-dripping, coating

## Abstract

A polyester fabric, coated with calcium alginate and nano-calcium borate composites (CAB-PL), was fabricated by a post-cross-linking method, with remarkable improvement of flame retardancy and thermal stability, as compared with the original polyester fabric (PL). The mechanical properties of CAB-PL and PL were studied, and characterizations and tests including Fourier transform infrared spectrum (FTIR), scanning electron microscopy (SEM), limiting oxygen index (LOI), cone calorimetry (CONE) and thermogravimetric analysis (TGA) were employed to evaluate the flame retardancy and thermostability. The test results of CAB-PL showed excellent mechanical strength and anti-dripping properties. In comparison with PL, TGA results indicate that the presence of surface-coated composites produced more char residue and can effectively inhibit the heat transmission, and the LOI value of CAB-PL was improved from 25 to 33. Moreover, CONE results show that 88.65% reduction of total smoke release (TSR) values was induced by the presence of CAB. In addition, the possible pyrolysis mechanisms for CAB-PL have been proposed based on the results of pyrolysis-gas chromatography–mass spectrometry (Py-GC-MS) analysis. The combined results can provide useful information for understanding the flame retardant mechanisms of alginates as well. In summary, polyester fabric was upgraded by coating it with the calcium alginate/nano-calcium borate, thus achieving extraordinary flame retardancy and thermal stability for various applications within the textile industry.

## 1. Introduction

In past decades, the production and application of textile materials have been significantly studied and great developments have been made. These materials have been of high interest because of their multiple advantages including low cost, excellent mechanical properties, and resistance to corrosion [1,2,3,4]. Polyester fabric, commonly known as its abbreviation—PET fiber—is a synthetic fiber spun from the polyester polycondensated from the organic dibasic acid and dihydric alcohol. It was invented in 1941 and developed as the first large variety of synthetic fiber. Its use has grown rapidly and is widely used in numerous fields such as home textiles, apparels, automobile seats, dyeing industry, tire production and etc. [5,6,7,8,9]. The global market capitalization for polyester fabric reached almost 100 billion dollars in 2017, and the compound growth rate was over 6% since 2012, reported by Transparency Market Research Inc. However, as a thermoplastic polymer, as with most other textiles, polyester fabric can be easily melted, decomposed, and even burnt when heated, making it a potential threat to human life due to its low flame retardancy during fire accidents [10]. Moreover, another serious safety issue is that the accompanying dripping problem of burning polyester fabric can be liable to ignite other polymeric materials, accelerate flame propagation, and finally cause expansion of the fire scale. Thus, improving the flame-retardant properties of polyester fabric is of great importance in view of its vast application, despite its low safety [11,12,13].

Recently, applications of multiple methods have been reported to improve the flame retardancy and thermal stability of textile materials. The direct addition of flame retardants during melt spinning, which facilitates effective grafting or coating on the fiber surface by adsorptive deposition, adhesion or chemical bonding, have been the most widely used [14,15,16,17,18]. The flame-retardant treatment of polyamide 66 fabric using a layer-by-layer method has been reported [19]. Boehmite nanoparticles have been applied to improve the flame-retardant properties of polyester fabric [20]. However, limitations such as poor compatibility and severe pollution are troublesome for these solutions. Furthermore, the process of fabric spinning can be easily affected, such that heavy smog and toxic gas may be released [21,22,23]. Therefore, developing an efficient and eco-friendly polyester fabric with flame retardant and anti-dripping properties still remains a challenge [24,25,26].

Surface coating is an important technology for improving flame retardancy without affecting the intrinsic properties of materials. It can be easily used with a variety of surfaces [27]. Paperboard has been coated with naturally biodegradable biopolymers to achieve extraordinary barrier performance for various applications within the packaging industry [28]. Polyvinylsilsesquioxane and ZnO nanoparticles has been combined as functional coatings for cotton fabric [29]. It was found that the functional cotton fabric showed excellent ultraviolet shielding and stable superhydrophobic properties. However, few reports on the flame-retardant treatment of polyester fabric via surface coating have been presented.

As a kind of natural polysaccharide, alginate is constituted from β-D-mannuronic acid (M block) and α-l-guluronic acid (G block) which are connected with 1,4-glycosidic bonds [27,28]. In the presence of Ca^2+^, the alginate forms calcium alginate, which is an insoluble gel and possesses inherent flame retardancy [29,30,31]. In a previous study, calcium alginate composites were prepared by introducing calcium borate nanoparticles using an in situ method. Studies suggested that the inorganic nano-microparticles can efficiently enhance the flame retardancy and thermal stability of the polymers [32,33,34,35]. For this study, the polyester fabric was treated with calcium alginate/calcium borate composites by combining surface-coating with post-cross-linking. The preparation is environmentally friendly and facile. The mechanical, flame-retardant and thermostablility properties of the fabricated materials were evaluated and pyrolysis mechanisms proposed based on the experimental data.

## 2. Materials and Methods

### 2.1. Materials

Sodium alginate was obtained from Guangfu Fine Chemical Research Institute (Tianjin, China). Borax and calcium chloride were purchased from Kermel Chemical Reagent Co., Ltd. (Tianjin, China). All the other reagents were obtained from Sinopharm Chemical Reagent Co., Ltd. (Shanghai, China) and were of analytical grade. Polyester fabric (300D × 300D) was provided by Huajin Co. Ltd. (Qingdao, China).

### 2.2. Preparation of Calcium Alginate/Nano-Calcium Borate Composites Coated Polyester Fabric

**Scheme 1:**
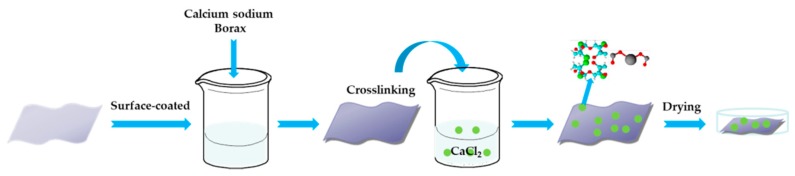
Preparation of calcium alginate/nano-calcium borate composites coated polyester fabric.

The preparation of the treated polyester fabric materials is shown schematically in Scheme 1. Briefly, 1.25 g of borax was dissolved in 250 mL of distilled water, and then 10 g of sodium alginate was slowly added into the borax solution to form a transparent gel while the solution was kept stirring at 70 °C for 5 min to ensure that the sodium alginate was dissolved completely. Then 10 pieces of the polyester fabric (PL) specified in 100 mm × 100 mm × 0.2 mm was coated with the obtained gel until its weight was three times the original. Then the coated fabric was immersed in 70% (m/v) calcium chloride solution until the gel shape was fixed. The pH of the solution was adjusted to 10 and the solution was kept at 30 °C for 10 h. Finally, the fabric was washed with distilled water, and dried at 55 °C to obtain the calcium alginate/nano-calcium borate composites coated polyester fabric (CAB-PL). The weight of the CAB-PL was increased by 8.6% to 12.790 g compared with the PL originally weighed 11.775 g.

### 2.3. Measurements and Characterizations

#### 2.3.1. Tensile Strength and Elongation

The tensile strength and elongation of the CAB-PL and PL was measured by employing an electronic fabric strength tester (YG(B) 026D–250, Darong Textile Instrument Co., Ltd., Wenzhou, China) according to ISO5081. The fabric materials, with the dimension of 100 mm × 50 mm, were balanced at room temperature and a relative humidity of 60% for 24 h prior to the tests.

#### 2.3.2. Scanning Electron Microscopy (SEM)

The morphology and microstructure of the PL, CAB-PL and their residues after heating in a quartz tube furnace (MXG1200–60, Micro-X Furnace Co., Ltd., Shanghai, China) were studied using SEM (JSM 6390LV, JEOL Inc., Tokyo, Japan). All the samples were sputtered with gold to avoid charging under the electron beam prior to the tests.

#### 2.3.3. Fourier Transform Infrared (FTIR)

FTIR spectroscopy (NICOLET iS50, Thermo Fisher Scientific Inc., Waltham, MA, USA) was employed to determine the chemical bonds in the PL and CAB-PL within the wavenumber range of 4000–500 cm^−1^.

#### 2.3.4. Limiting Oxygen Index (LOI)

A JF-3 type instrument (Jiangning Analytical Instrument Co., Nanjing, China) was employed to perform the LOI tests in accordance with the standard GB 5454–85 (China). And the dimensions of the test samples were 100 mm × 40 mm × 2 mm.

#### 2.3.5. Vertical Burning Rate (UL-94)

A vertical burning test instrument (CZF-2, Jiangning Analytical Instrument Co., Nanjing, China) was employed to perform the UL-94 tests according to ISO 5660 with the sheet dimensions of 130 mm × 13 mm × 2 mm.

#### 2.3.6. Cone Calorimeter (CONE)

A cone calorimeter device (M1354, Fire Testing Technology Ltd., East Grinstead, UK) was performed to measure the combustion behavior of the prepared samples according to ISO 5660. An external heat flux of 35 Kw/m^2^ was applied. The dimensions of the samples were 100 mm × 100 mm × 2 mm, and the samples were wrapped in aluminum foil and placed horizontally in a box with the identical dimension.

#### 2.3.7. Thermogravimetric Analysis (TGA)

TGA was performed under N_2_ atmosphere using a thermogravimetric analyzer (TG 209, NETZSCH Geraetebau GmbH, Selb, Germany), and a heating rate of 10 °C/min from room temperature to 800 °C was applied.

#### 2.3.8. Pyrolysis-Gas Chromatograpgy-Mass Spectrometry (Py-GC-MS)

To investigate on the pyrolysis products of the CAB-PL, Py-GC-MS was performed on a fast pyrolysis analyzer (CDS 5200, CDS Analytical Inc., Oxford, UK) coupled with a GC-MS (Clarus 680GC-SQ8MS, PerkinElmer, Inc., Shelton, CT, USA). A pyrolysis temperature of 700 °C and carrier gas of He was applied. The GC temperature program was set to start at 50 °C and then increased to 300 °C within 15 min. The products were identified using the characteristic GC/MS spectra, by comparison with the National Institute of Standards and Technology (NIST) library and other reports.

## 3. Results and Discussion

### 3.1. Tensile Strength and Elongation

**Figure 1:**
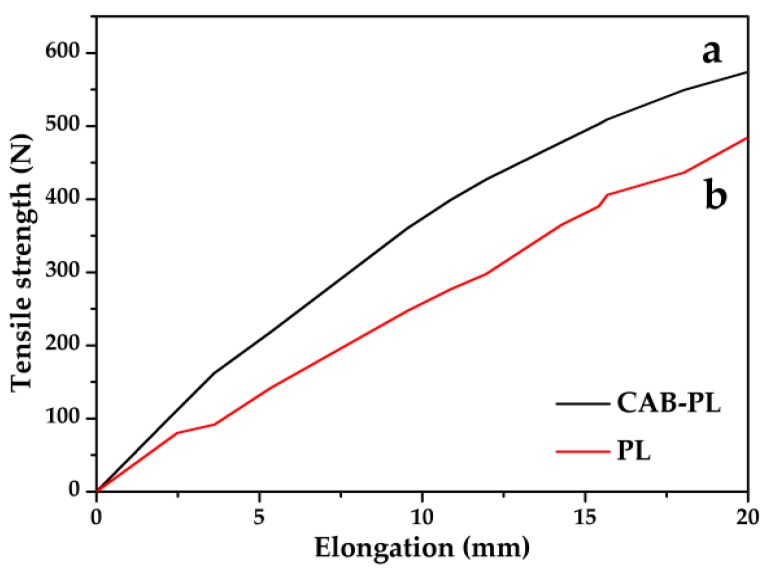
Tensile strength–elongation curves of the (**a**) polyester fabric coated with calcium alginate /nano-calcium borate composites (CAB-PL) and (**b**) polyester fabric (PL).

Figure 1 shows the tensile strength–elongation curves of the CAB-PL and PL, and the related data are displayed in Table 1. The tensile strength of the PL was 518 N, while, comparatively, it was increased by 14% to 592 N for the CAB-PL, which can be attributed to the uniform dispersion of the composites on the fabric surface. The results suggest that the incorporation of the composites into the PL exerts remarkable effects on the enhancement of the tensile strength. However, as seen from Table 1, the elongation at the break of the CAB-PL does not show noticeable improvements compared to the PL, which may be due to the insufficient flexibility of the PL leading to occurrence of instantaneous fracture during the tests.

### 3.2. SEM

**Figure 2:**
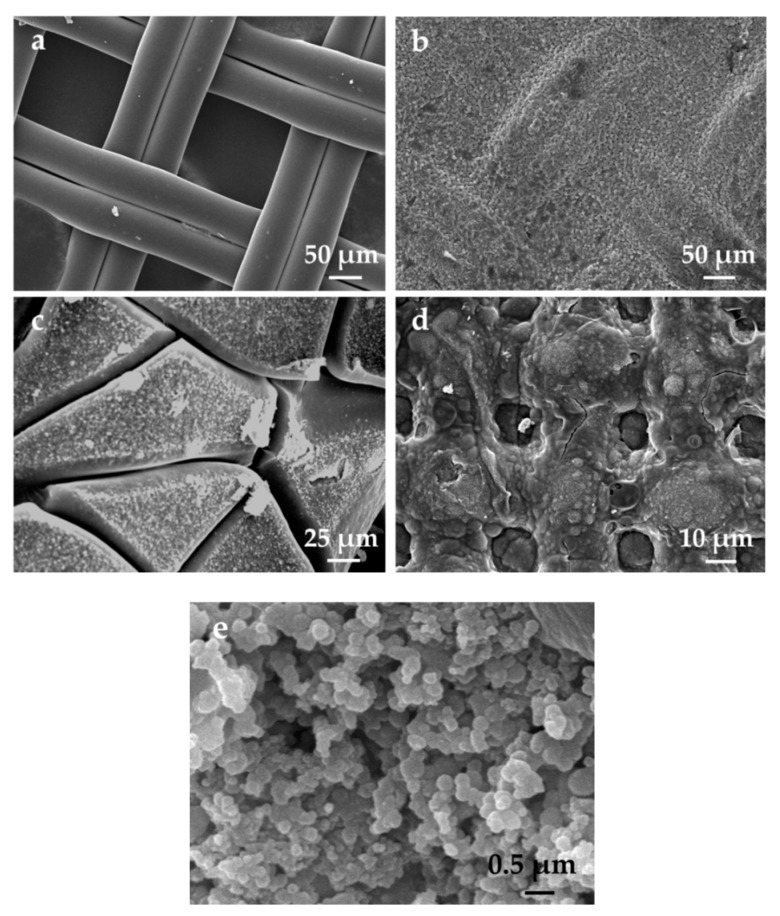
SEM images of the (**a**) PL, (**b**) CAB-PL, (**c**) PL heated at 700 °C, (**d**) CAB-PL heated at 700 °C and (**e**) nano-calcium borate composites coated on the surface of polyester fabric.

The SEM images of the PL, CAB-PL and their residues produced from calcining at 700 °C are presented in Figure 2. It can be observed from Table 2a that the surface of the PL was smooth and filled with a unique grid. And for the CAB-PL, as seen in Figure 2b,e, the CAB composites were successfully coated on the surface of the PL with uniform distribution, and the spherical nanoparticles of calcium borate can be observed in the composites. As shown in Figure 2c,d, notable morphological changes can be seen in the residues produced while calcining at 700 °C from both the prepared samples. And it can be observed that there were more cracking residues on the surface of the PL than that of the CAB-PL. Meanwhile, some grids of the CAB-PL still remained, indicating that the calcium alginate/nano-calcium borate composites were degraded while the residue layer was produced to protect the surface of the materials.

### 3.3. FTIR Analysis

**Figure 3:**
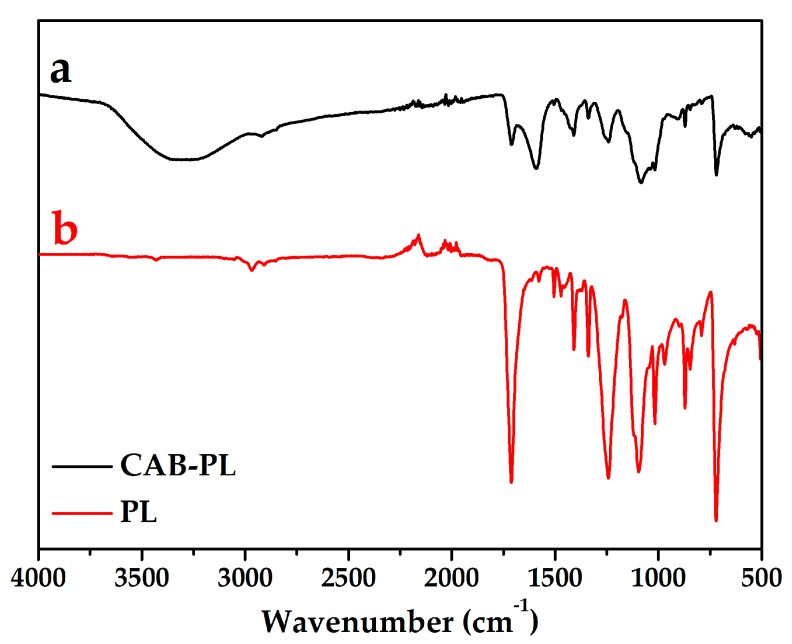
FTIR spectra of the (**a**) CAB-PL and (**b**) PL.

FTIR spectroscopy was employed to investigate the structures of the CAB-PL and PL, and the results are shown in Figure 3. For the spectrum of the PL, the peak at 1713 cm^−1^ was corresponding to the C=O group of aromatic ester, while the peaks at 1087 cm^−1^ and 1242 cm^−1^ can be assigned to the ester C=O stretching vibration [36]. And the peaks at the range of 1403 cm^−1^ –1509 cm^−1^ were ascribed to the C=C stretching vibration. The characteristic bands at 974 cm^−1^ and 1022 cm^−1^ were contributed to the C–O stretching in-plane vibration. Compared to the PL, the FTIR spectrum of the CAB-PL showed several changes: the new peaks appeared at 3310 cm^−1^ and 1585 cm^−1^ can be ascribed to the –OH and C–O–C stretching vibration present in calcium alginate, meanwhile the bands at 1338 cm^−1^ and 1408 cm^−1^ were corresponding to the asymmetric stretching of B–O, and the peak at 933 cm^−1^ can be assigned to the B–O symmetric stretching [37]. The results indicate the existence of the calcium alginate/nano-calcium borate composites in the CAB-PL.

### 3.4. Flame Retardancy and Combustion Behavior of the CAB-PL and PL

#### 3.4.1. LOI

LOI refers to the volume fraction concentration of oxygen when it can exactly support the sample combustion under oxygen-nitrogen atmosphere. It is an important index that characterizes the burning behavior of tested materials. The LOI test results of the CAB-PL and PL are listed in Table 1. It can be noticed that the LOI value of the CAB-PL was 34, which was remarkably higher than that of the PL (25), indicating the CAB-PL was more difficult to be combusted. Moreover, the LOI value of the CAB-PL was much higher than 27, indicating it can be considered as a reliable flame retardant material. The results reflect the important contribution of introduction of the composites to the PL for the improvement of flame retardant properties.

#### 3.4.2. UL-94

As an important index to evaluate the flame retardancy of materials, UL-94 test has been widely used for the evaluation of combustion behavior. The UL-94 results of the prepared two samples are depicted in Table 2. It can be seen that the PL was completely consumed during the test, while the burning tendency of the CAB-PL was notably reduced, comparatively. As seen from Table 2, it can be noted that the UL-94 rating of the CAB-PL was V-0. When the PL was ignited, it showed a state of intense burning, while only weak flame can be observed for the CAB-PL. Moreover, the after-flame time of the PL and CAB-PL was 3.9 s and 0 s, respectively, suggesting that the CAB-PL can be self-extinguishing after being ignited. It can be inferred that the flame retardancy of the PL was improved after being coated with the composites. Moreover, it can be observed from the images in Table 2 that the CAB-PL showed excellent anti-dripping properties, in comparison with the PL. The results indicate that the composites were deposited ahead of the fabric. And especially at the beginning of the combustion, the produced residues from the composites provided supports for the fabric and prevented fire from spreading.

#### 3.4.3. CONE

**Figure 4:**
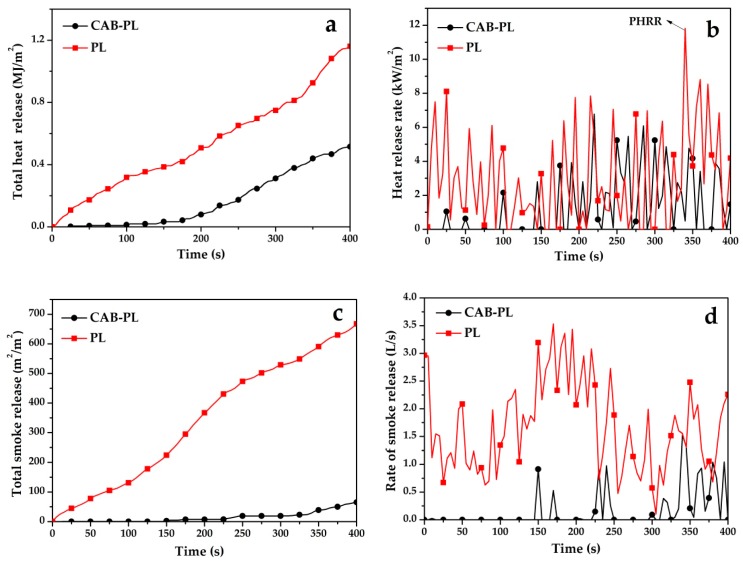
(**a**) Total heat release (THR), (**b**) heat release rate (HRR), (**c**) total smoke release (TSR), and (**d**) rate of smoke release (RSR) curves of the CAB-PL and PL changing over time.

The cone calorimetry test simulates the real flame scene, and the obtained test data can evaluate the behavior of the material during fire, such as total heat release (THR), heat release rate (HRR), total smoke release (TSR) and etc. The CONE results of the CAB-PL and PL are shown in Figure 4 and Table 1. The THR curves of the two materials are depicted in Figure 4a, and it can be seen that, for the PL, the THR values showed a rapid decrease after being ignited and finally reached 1.7 MJ/m^2^ at the end of burning, while the CAB-PL was burned slowly at the beginning stage of the combustion with a much lower THR value of 0.6 MJ/m^2^. Namely, there was 61.8% reduction of THR value for the CAB-PL, compared to that of the PL. As the heat released by the combustion of materials can raise surrounding temperature during burning, a lower THR value can be beneficial to materials. HRR is another important index to explore the combustion behavior of materials, and the HRR curves of the two prepared samples are presented in Figure 4b [38]. Both of the samples showed intermittent release heat, indicating that the materials were non-persistently exothermic during ignition. Meanwhile, the HRR was increased with burning time. Moreover, the peak heat release rate (PHRR) values for the CAB-PL and PL reached 6.8 Kw/m^2^ and 11.8 Kw/m^2^ at the end of burning, respectively, indicating significant reduction of flammability for the CAB-PL. The results reflect that the composites can allow the coated polyester fabric to suppress flame propagation and further reduce the possibility of fire accidents.

As smoke is the major factor to cause death during fire accidents, TSR and rate of smoke release (RSR) values are important parameters to measure the safety of materials [39]. It can be noted from Figure 4c that the CAB-PL showed almost no smoke release during the combustion and a flat curve was observed before 150 s, however, for the PL, plenty of smoke was continuously produced over time during the tests. Meanwhile, as seen in Figure 4d, the RSR corresponding to the HRR curve exhibited intermittent release, and the curve of the PL was higher than that of the CAB-PL. Furthermore, the related indexes from the tests significantly decreased as the composites were coated on the PL. For instance, the two samples showed a great gap at the final stage: the TSR value of PL was 757 m^2^/m^2^ while that of the CAB-PL was only 86 m^2^/m^2^ after burning for 400 s. The 88.7% reduction of the TSR values between the two materials indicates better safety performance of the CAB-PL, in comparison with that of the PL.

From the results of CONE, it can be noted that the CAB-PL released little heat and smoke, indicating its excellent flame-retardant behavior in real fire hazards. The highly efficient flame retardancy of the CAB-PL inferred from the experimental data can be attributed, as follows, to: on the one hand, the CAB-PL possessed inherent flame retardancy; on the other hand, the composites can produce char layer to shelter the fabrics from the oxygen and heat while heating. Thus, in an actual fire, especially for the indoor environment, CAB-PL can inhibit the spread of the flame to guarantee the safety of the architecture and also provide more saving time for firemen during the combustion.

#### 3.4.4. TGA and Differential Thermogravimetry (DTG)

**Figure 5:**
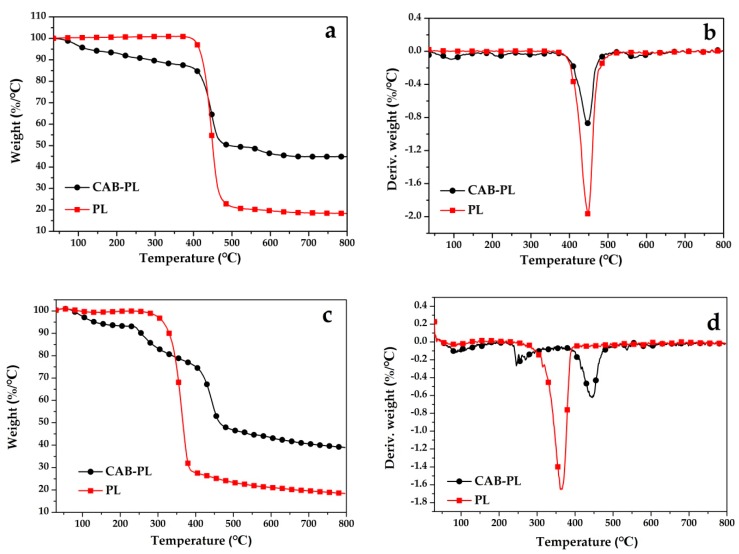
(**a**) TG and (**b**) DTG curves of the CAB-PL and PL under air; (**c**) TG and (**d**) DTG curves of the CAB-PL and PL under N_2_.

TGA was performed to investigate the thermal stability of the CAB-PL and PL, and the TG and DTG curves are displayed in Figure 5. It can be easily noticed that the PL appeared a rapid weight loss with one main step at around 400 °C–500 °C, while the CAB-PL showed three steps of degradation. However, in comparison with the PL, the CAB-PL exhibited a lower initial degradation temperature, which may be due to evaporation of the physical moisture in the composites. The second step weight loss of the CAB-PL started at 400 °C, which can be mainly attributed to the breaking of glycosidic bonds and the loss of crystal water in the calcium borate. At the third stage, the two materials were completely decomposed. Obviously, the CAB-PL showed a higher remnant weight than that of the PL at the temperature ranging from 450 °C to 800 °C. This may be the reason that the composites decomposed ahead of the PL and the formation of the char residue provided support to the PL.

The TG curves of the CAB-PL and PL in air atmosphere are presented in Figure 5c, which are different from those tested in N_2_ atmosphere. Two main stages are observed in the thermal oxidative degradation of the two samples. For the PL, the weight loss between 275 °C and 380 °C can be attributed to breaking of the main chain from the polyester, while some small molecules were released including H_2_O, CO_2_, hydrocarbon fragments and etc. And the next step, started at 380 °C, can be assigned to the thermal-oxidative degradation of the residues. For the CAB-PL, at first it was similar to the TG results under N_2_. And before 350 °C, its curves were lower than that of the PL, showing more initial weight loss, which may be attributed to the evaporation of the physical moisture in the composites, or the decomposition of the calcium alginate. The degradation of the composites at the lower temperature can be beneficial, because the formation of char can be initiated in the early stage of the fire accidents, preventing further combustion of the polyester and stopping the flame from spreading.

Besides, it can be seen that the coated fabric showed wider degradation temperature range between 350 °C and 495 °C. And the maximum weight loss of the CAB-PL appeared at 450 °C while the PL showed at 365 °C, as shown in Figure 5d. The results indicate that the CAB-PL had lower degradation speed and longer thermal oxidative degradation time, comparatively.

By comparing the results in Figure 5a,c, it can be noticed that the CAB-PL showed more weight loss in air than that in N_2_ when the decomposition was completed. And the weight loss of the CAB-PL in the air was similar with that in N_2_ before 250 °C; thus, it can be inferred that the oxygen started to participate in the degradation after 250 °C. In addition, the combined results suggest that the residues were relatively stable once they were formed under both gas atmospheres.

### 3.5. Py-GC-MS

**Figure 6:**
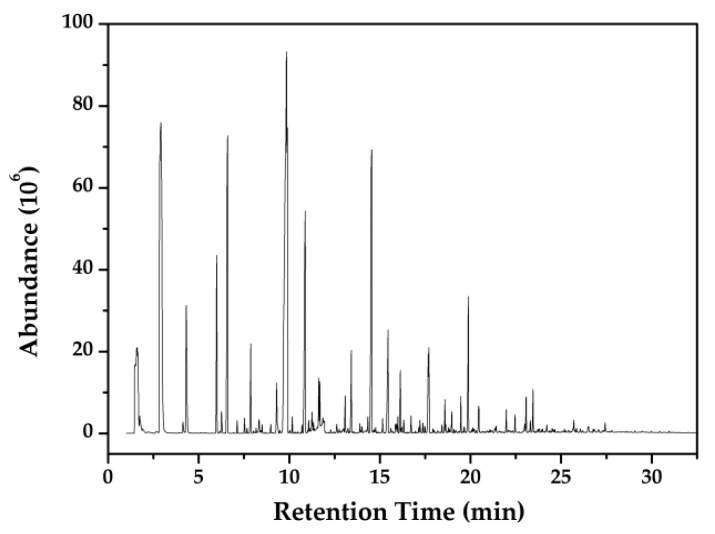
Py-GC-MS spectrum of the CAB-PL at 700 °C.

Py-GC-MS tests were performed to analyze the composition of gaseous production in the pyrolysis process of the CAB-PL. According to the comparison with the NIST library, the major characteristic compounds are summarized and their structures are shown in Table 3. And Figure 6 depicts the total ion intensity of gases formed in the CAB-PL.

As seen from Figure 6 and Table 3, the main compounds are as follows: carbon dioxide, 2-hexen-4-yne, 2-methylbenzyl alcohol, 2-ethenyl-naphthalene, annulene, benzaldehyde etc., and the yield of those is 63.9% in total. Three types of pyrolysis gases were produced from the CAB-PL: one was the chained small molecule compounds, including the carbon dioxide, 2,3-butanedione and etc.; the second type involved products with low molecular weight containing a benzene ring, for instance, methylbenzene, 1,3-dimethylbenzen and etc.; the third type contained polycyclic carbon compounds included indene, naphthalene, 4-phenylacetophenone, and etc.

**Figure 7:**
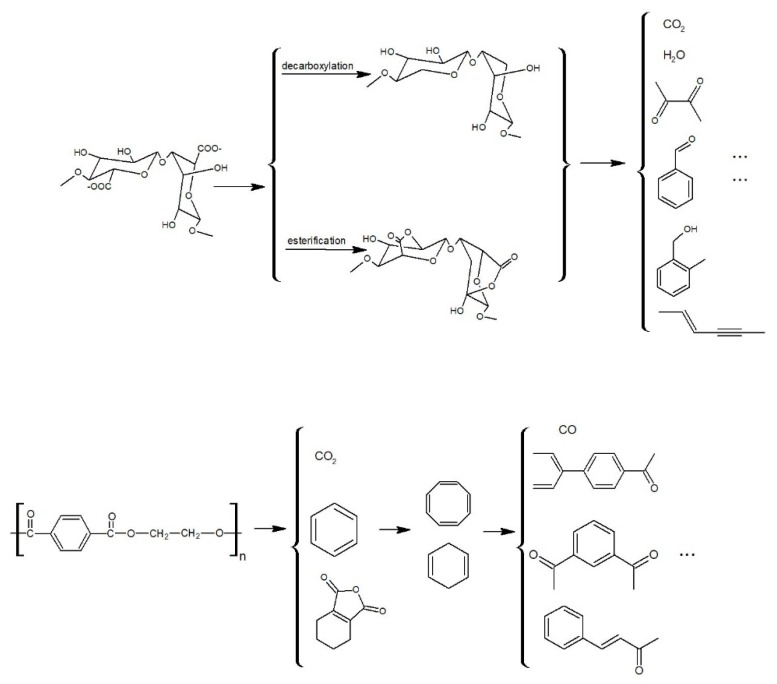
Proposed pyrolysis mechanisms of the CAB-PL.

On the basis of the experimental data, the possible pyrolysis mechanisms of the CAB-PL are proposed and depicted in Figure 7. The decomposition of the CAB-PL was consisted of the calcium alginate/nano-calcium borate composites and polyester fabric both. For the composites, dehydration occurred at the first step and evaporation of H_2_O reached maximum at the next step of thermal degradation. Then the breaking of the glycosidic bonds, esterification and decarboxylation were in succession, which released the small molecule compounds (CO_2_, H_2_O, and etc.) and produced compounds such as 2,3-butanedione, phenylpropiolic acid, benzaldehyde and so on.

The decomposition mechanism of polyester cloth coated with the composite materials was also analyzed. Under an external heat source, the PL began to become soft when reached a certain temperature, and was further melted. At the early stage of heating, cyclic oligomer was formed in the molecule, while carboxylic acid and vinyl ester were also formed, and soon, terephthalic acid was generated and finally decarboxylated to produce acid anhydride and carbon dioxide or benzene, etc. The cyclic olefin cross-linking structure was formed by the polymerization reaction and the chain scission process, and the small molecule ketone, carbon monoxide and etc. were directly formed by the degradation.

## 4. Conclusions

Polyester fabric with flame retardant and anti-dripping properties was prepared by a simple surface-coating process with the calcium alginate/nano-calcium borate composites, combined with a green post-cross-linking method. The combustion performance and pyrolysis mechanism of the coated fabric were studied. The results show that the flame retardant and anti-dripping properties of the coated composites were effectively improved, compared to the basic polyester fabric. During the decomposition process, as the composites covered the surface of the fabric to shelter the material from heat, the coated fabric showed excellent thermal stability. Moreover, in the pyrolysis process, the coated fabric formed more stable compounds with ring structures, which provided useful information to understand its pyrolysis mechanism. The study develops the inherent flame retardancy of the calcium alginate for textile industry and enlarges the application fields of the polyester fabric. It is intended to be a useful incremental work with respect to previous work, and the designed strategy can guide developing advanced functional textile in future work, which may likely be found in the advanced protective textiles.

## Figures and Tables

**Table 1:** Mechanical properties and combustion behavior of the CAB-PL and PL.

Samples	Tensile Strengh (N)	Elongation at Break (%)	LOI (%)	PHRR (Kw/m^2^)	TSR (MJ/m^2^)
CAB-PL	592	26.9	34	6.8	86
PL	518	26.2	25	11.8	757

**Table 2:** Results of UL-94 for CAB-PL and PL.

Samples	CAB-PL		PL	
Print cloth	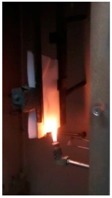	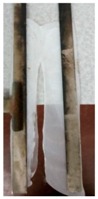	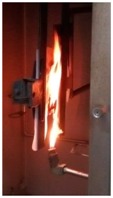	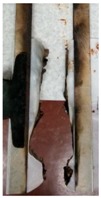
After flame time (s)	0		3.9	
After glow time (s)	0		0	
Dripping	No		Yes	
Rate	V-0		N.R	

Note: N.R. stands for no rating.

**Table 3:** Pyrolysis products of the CAB-PL at 700 °C.

S.N.	Molecular Structure	Name of Compound	Time	Area
1	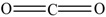	carbon dioxide	1.55	3.05
2	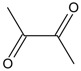	2,3-butanedione	1.62	3.94
3	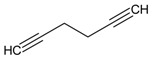	1,5-hexadiyne	2.85	4.78
4	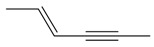	2-hexen-4-yne	2.91	14.56
5	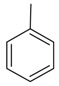	methylbenzene	4.32	3.17
6	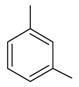	1,3-dimethylbenzen	5.99	3.10
7	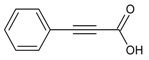	phenylpropiolic acid	6.26	0.39
8	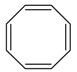	annulene	6.59	7.90
9	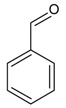	benzaldehyde	7.87	7.68
10	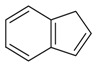	indene	9.31	0.72
11	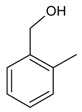	2-methylbenzyl alcohol	9.84	21.90
12	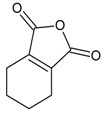	1,3-isobenzofurandione	9.89	5.60
13	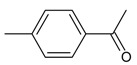	1-(4-methylphenyl)- acetophenone	11.63	0.65
14	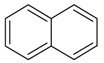	naphthalene	11.68	0.57
15	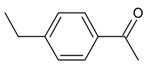	1-(4-ethylphenyl)- acetophenone	13.08	0.54
16	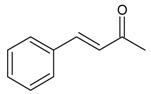	4-benzalacetone	13.42	1.32
17	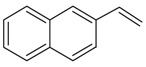	2-ethenyl-naphthalene	14.55	8.87
18	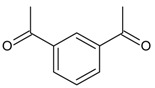	1,3-diacetylbenzene	15.45	3.32
19	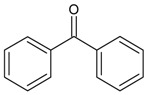	benzophenone	17.67	1.53
20	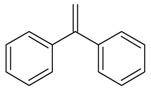	1,1′-diphenylethylene	17.71	1.43
21	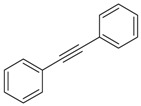	diphenylacetylene	19.46	0.49
22	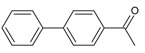	4-phenylacetophenone	19.89	2.85
23	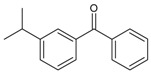	(3-isopropylphenyl) (phenyl) methanone	21.98	0.33
24	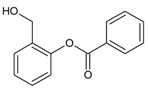	(3-isopropylphenyl) (phenyl) methanone	22.45	0.27
25	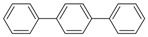	p-terphenyl	23.45	0.57
